# Quantitative analysis of similarity between cerebral arterial blood volume and intracranial pressure pulse waveforms during intracranial pressure plateau waves

**DOI:** 10.1016/j.bas.2024.102832

**Published:** 2024-05-07

**Authors:** Arkadiusz Ziółkowski, Magdalena Kasprowicz, Agnieszka Kazimierska, Marek Czosnyka

**Affiliations:** a Department of Biomedical Engineering, Faculty of Fundamental Problems of Technology, Wroclaw University of Science and Technology, Wroclaw, Poland; b Division of Neurosurgery, Department of Clinical Neurosciences, Addenbrooke's Hospital, University of Cambridge, Cambridge, United Kingdom; c Institute of Electronic Systems, Faculty of Electronics and Information Technology, Warsaw University of Technology, Warsaw, Poland

**Keywords:** Intracranial pressure, Cerebral blood flow velocity, Transcranial Doppler, Morphological analysis, Pulse shape analysis, Brain blood circulation

## Abstract

**Introduction:**

Both intracranial pressure (ICP) and cerebral arterial blood volume (C_a_BV) have a pulsatile character related to the cardiac cycle. The evolution of the shape of ICP pulses under increasing ICP or decreasing intracranial compliance is well documented. Nevertheless, the exact origin of the alterations in the ICP morphology remains unclear.

**Research question:**

Does ICP pulse waveform become similar to non-invasively estimated C_a_BV pulse during ICP plateau waves.

**Material and methods:**

A total of 15 plateau waves recorded in 15 traumatic brain injured patients were analyzed. C_a_BV pulse waveforms were calculated using global cerebral blood flow model from transcranial Doppler cerebral blood flow velocity (CBFV) signals. The difference index (DI) was used to quantify the similarity between ICP and C_a_BV waveforms. DI was calculated as the sum of absolute sample-by-sample differences between ICP and C_a_BV waveforms, representing the area between the pulses.

**Results:**

ICP increased (19.4 mm Hg [Q1–Q3: 18.2–23.4 mm Hg] vs. 42.7 mm Hg [Q1–Q3: 36.5–45.1 mm Hg], p < 0.001) while CBFV decreased (44.2 cm/s [Q1–Q3: 34.8–69.5 cm/s] vs. 32.9 cm/s [Q1–Q3: 24.7–68.2 cm/s], p = 0.002) during plateau waves. DI was smaller during the plateau waves (20.4 [Q1–Q3: 15.74–23.0]) compared to the baselines (26.3 [Q1–Q3: 24.2–34.7], p < 0.001).

**Discussion and conclusion:**

The area between corresponding ICP and C_a_BV pulse waveforms decreased during the plateau waves which suggests they became similar in shape. C_a_BV may play a significant role in determining the shape of ICP pulses during the plateau waves and might be a driving force in formulating ICP elevation.

## Highlights

• During plateau waves ICP pulses change from triphasic to more triangular shape shape
• ICP waveforms are more similar to C_a_BV pulses during plateau waves than at baseline.
• During plateau waves pulse C_a_BV may be transmitted directly to intracranial pressure signal.
• The C_a_BV pulse may have a dominant impact on the shape of ICP pulse waveform during plateau wave.
• Changes in C_a_BV may be a driving force behind the formulation of ICP elevation.

## 1 Introduction

**Fig. 1:**
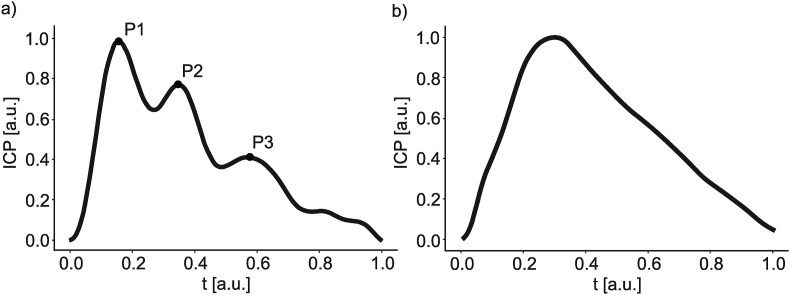
Illustrative examples of the shapes of intracranial pressure (ICP) pulse waveform: a) normal saw-tooth shape with three visible peaks (P1, P2, P3), b) pathologically rounded shape without indistinguishable peaks P1 and P3. The pulses are presented as normalized in terms of duration and pressure level. a.u. - arbitrary units.

The normal intracranial pressure (ICP) pulse waveform is often characterized by three local maxima known as peaks P1, P2, and P3 (see Fig. 1a) (Cardoso et al., 1983; Dunbar et al., 1966; Germon, 1988). The first peak (P1) is associated with the systolic peak of both arterial blood pressure (ABP) and cerebral blood flow velocity (CBFV) pulses while the second peak is linked with the maximum of cerebral arterial blood volume pulse (Carrera et al., 2010). The exact origin of the third peak (P3) is not clearly understood; however, it is hypothesized that it may be related to venous blood outflow (Czosnyka and Czosnyka, 2020).

The evolution of the shape of the ICP pulse waveform under the conditions of increasing ICP and/or decreasing compliance is relatively well known (Kazimierska et al., 2021; Mataczynski et al., 2022; Nucci et al., 2016). Normally, P1 dominates the waveform, giving it a saw-tooth appearance (Fig. 1a). However, as mean ICP increases and/or compliance decreases, the amplitudes of the characteristic peaks change. First, P2 and P3 exceed P1, and then the waveform gradually becomes rounded or triangular, with undistinguishable P1 and P3 (Fig. 1b) (Cardoso et al., 1983; Chopp and Portnoy, 1980; Contant et al., 1995). This observation led to the idea that the shape of ICP pulse waveform can be grouped into characteristic classes, ranging from a normal triphasic shape (class I) to a pathologically rounded shape (class IV) (Mataczynski et al., 2022; Nucci et al., 2016).

The cerebral arterial blood volume (C_a_BV) signal is also pulsatile but there is currently no consensus about the shape of C_a_BV pulse waveform. In studies where C_a_BV was measured using an experimental ultrasound-based method, the shape of the C_a_BV pulse was reported to have a saw-tooth appearance with three identifiable peaks (Chambers et al., 2005). On the other hand, studies using magnetic resonance imaging (Vallet et al., 2020; Yiallourou et al., 2015) or a mathematical model of global cerebral blood flow (Carrera et al., 2010; Czosnyka and Czosnyka, 2020; Kim et al., 2009; Uryga et al., 2019) reported a more rounded shape, either with only two visible peaks or without clearly distinguishable peaks. It has been suggested that during specific vasogenic episodes, such as ICP plateau waves, the ICP pulse amplitude is strongly associated with the amplitude of C_a_BV waveform (Carrera et al., 2010). However, quantitative comparison between the shape of C_a_BV and ICP pulses during plateau waves has not been performed to date. The ICP plateau wave is associated with a significant increase in mean ICP, often reaching high levels above 40 mm Hg (Rosner and Becker, 1984), increased compliance of the arterial part of the cerebral vascular bed, reduced cerebrospinal compliance, and the occurrence of pathologically altered shapes of the ICP pulse waveform (Kim et al., 2009). The important contributing cause of these changes is the cerebral vasodilatory cascade (Rosner's cascade) in response to a sudden drop in ABP (vasodilatory stimulus). This leads to a rapid increase in cerebral blood volume (Hayashi et al., 1985), resulting in elevated ICP and decreased cerebral perfusion pressure (CPP). Subsequent vasodilation further elevates ICP, continuing this cycle until maximum vasodilation is achieved. A vasoconstrictive stimulus interrupts this process, reversing the cascade, initiating a vasoconstrictive cycle, and restoring normal ICP levels (Rosner and Becker, 1984). Therefore, we hypothesize that the C_a_BV pulse may have a dominant impact on the ICP pulse waveform and the ICP pulse becomes similar to the shape of C_a_BV pulse waveform during ICP plateau wave.

## 2 Material and methods

### 2.1 Patient cohort

We retrospectively analyzed high-resolution recordings of ICP, CBFV, and ABP from a database of 345 adult traumatic brain injured (TBI) patients who were hospitalized at Addenbrooke's Hospital in Cambridge, United Kingdom between 1992 and 2009. These patients were mechanically ventilated and under sedation. In the event that CPP fell below 60 mm Hg due to a decrease in ABP, fluid loading and dopamine injections (2–15 μg kg^−1^ min^−1^) were administered. If necessary, norepinephrine injections were given at a rate of 0.5 μg kg^−1^ min^−1^. To manage ICP levels above 20 mm Hg lasting more than 15 min, mannitol boluses were administered (200 ml of 20% solution over 20 min or longer) and ventilation volume was increased to achieve mild hypocapnia, with a partial pressure of arterial CO_2_ ranging from 28 to 35 mm Hg.

All signals were recorded in accordance with the routine clinical procedures for monitoring cerebral autoregulation (Czosnyka et al., 2001). This multimodal brain monitoring approach was approved by the user committee of the multidisciplinary Neurosciences Critical Care Unit (NCCU) prior to 1997. Subsequently, regional ethical committee approval (30 REC 97/291) was obtained for the recording of anonymized data. The study adhered to the principles outlined in the Declaration of Helsinki, and the data was fully anonymized to eliminate any risk of data protection issues.

### 2.2 Data acquisition

The M1 segment of the middle cerebral artery was selected for monitoring CBFV using Multi Dop X4 (DWL Elektronische Systeme, Sipplingen, Germany) or Neuroguard (Medasonic, CA, United States) transcranial Doppler (TCD) ultrasonography system with a 2-MHz probe. TCD monitoring was performed on the same side as the ICP probe insertion which was chosen on an individual basis for each patient. Each TCD recording was done by a single, experienced operator. ICP was monitored using intraparenchymal probes (Codman & Shurtleff, MA, United States or Camino Laboratories, CA, United States) while ABP was invasively monitored from the radial artery using pressure monitoring kits (Baxter Healthcare, CA, United States or Sidcup, United Kingdom). All signals were recorded concurrently at a 50 Hz sampling rate using an analog-to-digital converter (DT9801, Data Translation, Marlboro, Mass, United States) and waveform recording software WREC (W. Zabolotny, Warsaw University of Technology, Poland) or BioSAn (P. Smielewski, University of Cambridge, United Kingdom). ICP and ABP were monitored continuously over days, whereas CBFV was assessed at specific timepoints chosen by a clinician. Although the end-tidal CO_2_ level was controlled, it was not continuously monitored.

### 2.3 Data processing

During retrospective analysis performed using ICM+ software (Cambridge Enterprise Ltd., Cambridge, United Kingdom), plateau waves were identified based on the following criteria: an increase in mean ICP above 40 mm Hg, a relative increase in mean ICP equal to or greater than 15 mm Hg, and a decrease in CPP equal to or greater than 10 mm Hg for at least 3 min. The definition of plateau wave adhered as closely as possible to the criteria outlined in experimental research (Rosner and Becker, 1984), but also took into account the clinical approach to managing prolonged increases in ICP which results in smaller magnitude of changes observed in actively treated TBI patients.

**Fig. 2:**
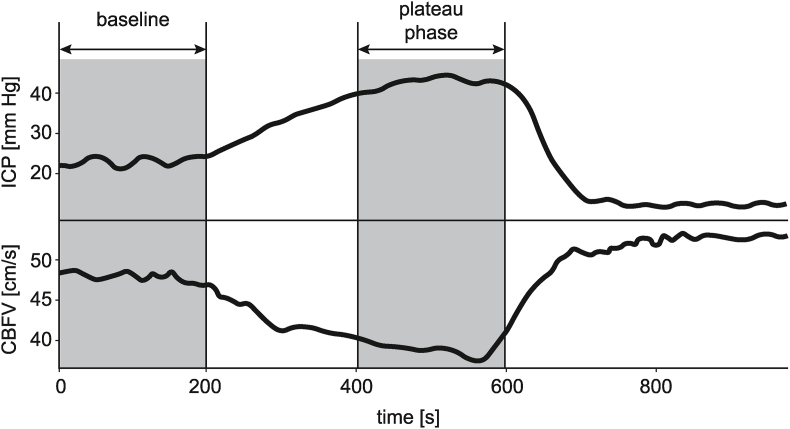
Illustrative example of mean intracranial pressure (ICP) and mean cerebral blood flow velocity (CBFV) signals recorded in a single patient. Gray areas indicate the periods selected as baseline and plateau phase of the plateau wave.

A total of 20 recordings with identified plateau waves were then visually inspected in ICM+ software to exclude signals that exhibited poor quality for most of the recording duration. During this process, 3 recordings were rejected due to low quality of the ICP signal and 2 recordings were excluded due to low quality of the CBFV signal. For further analysis, two periods were manually selected from each recording: approximately 4 min of high-quality signals that preceded the onset of an ICP plateau wave (referred to as the baseline period) and the entire duration of the plateau phase of the plateau wave which varied in length. The baseline period was identified as the segment of the recording before the increase in ICP associated with the plateau wave. The plateau period was defined as the part of the signal where the average ICP was equal to or greater than 90% of the maximum ICP value observed throughout the entire wave. An example of a recording with selected baseline and plateau period is presented in Fig. 2.

Prior to pulse shape similarity analysis, the ICP and CBFV signals were up-sampled to a frequency of 200 Hz using linear interpolation to improve their temporal resolution and to facilitate accurate detection of pulse onsets. A low-pass filter with a cutoff frequency of 12 Hz was applied to both signals to eliminate high-frequency noise. The modified Scholkmann algorithm (Bishop and Ercole, 2018) was used for pulse onset detection. All of the detected ICP and CBFV pulses were visually inspected using an application custom-written in Python to remove artifacts and distorted pulses (e.g. containing short-term disturbances or non-physiological values and peaks resulting from artifacts at the signal collection stage) from further analysis.

**Fig. 3:**
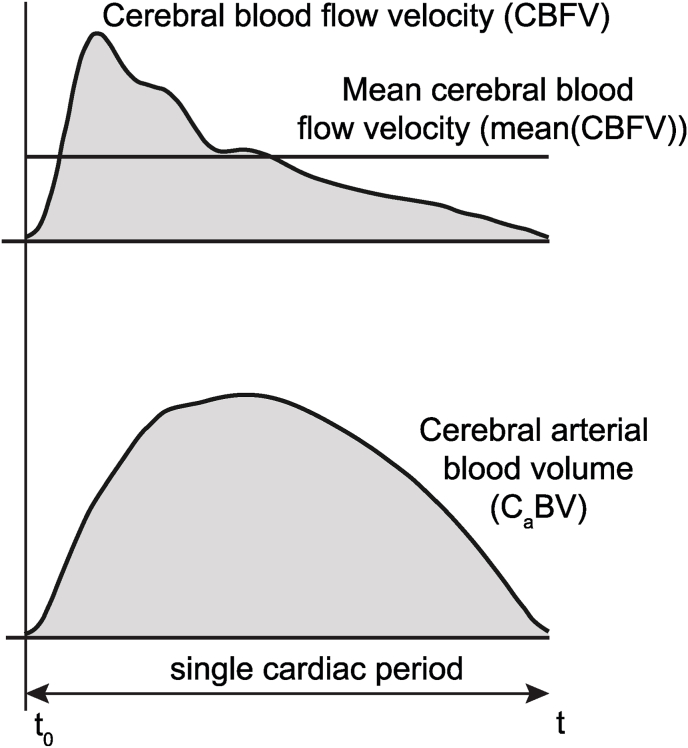
Visualization of cerebral arterial blood volume (C_a_BV) calculated over a single cardiac cycle using the constant flow forward model based on cerebral blood flow velocity (CBFV) signal recorded non-invasively in a healthy human (Kim et al., 2009). t_0_ – beginning of the cardiac cycle, t – end of the cardiac cycle.

The C_a_BV pulses were calculated from all not rejected CBFV pulses utilizing the constant flow forward (CFF) model of global cerebral circulation described in (Avezaat et al., 1986; Kim et al., 2009; Uryga et al., 2019). The model is based on the assumption that the change in cerebral blood volume during one cardiac cycle corresponds to the difference between arterial inflow and venous outflow (Avezaat and Eijndhoven, 1984). It further assumes that since venous outflow has low pulsatility compared to arterial inflow and exhibits negligible changes over the cardiac cycle (Aaslid et al., 1991), venous outflow can be approximated as the arterial inflow averaged in a longer window (usually 6 s). Finally, to replace direct cerebral blood flow measurement with non-invasively recorded CBFV, the model assumes that the cross-sectional area of the insonated artery remains constant during the recording. Consequently, changes in C_a_BV can be calculated as the time integral of the difference between pulsatile CBFV and mean CBFV (see Fig. 3). In this study, we estimated C_a_BV pulse waveforms from discrete digital recordings of CBFV according to equation (1):

(1) $${\Delta C}_{a}BV\left(n\right)=\sum_{i=1}^{n}\left[CBFV\left(i\right)-mean\left(CBFV\right)\right]\Delta t\;\left[cm\right]$$

where ΔC_a_BV(n) – change in cerebral arterial blood volume; n – the following number of samples from the beginning of one cardiac cycle, CBFV(i) – cerebral blood flow velocity at each time instant, mean(CBFV) – cerebral blood flow velocity averaged in a 6-s window, Δt – the time interval between two consecutive samples.

**Fig. 4:**
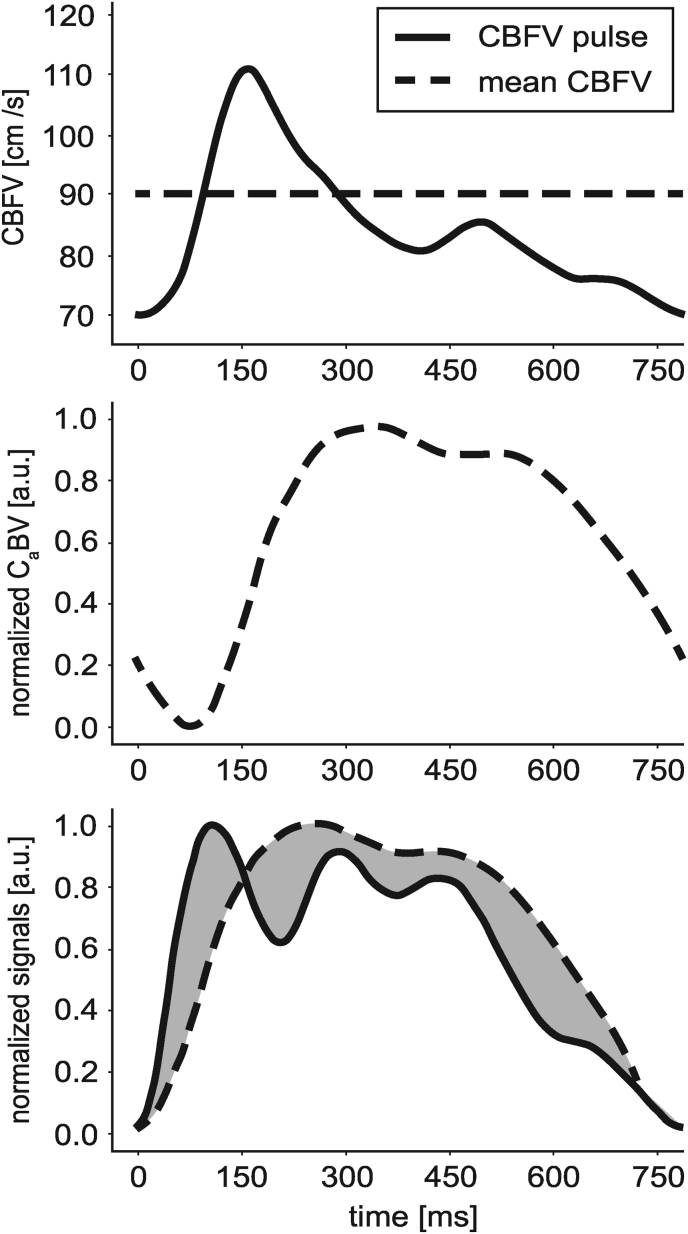
The top graph shows an example of the cerebral blood flow velocity (CBFV) pulse waveform used to calculate cerebral arterial blood volume (C_a_BV) pulse waveform (middle graph) based on a mathematical model of cerebral circulation (Kim et al., 2009). In the lower graph, the beginning of the C_a_BV pulse waveform is synchronized with the beginning of the corresponding intracranial pressure (ICP) pulse waveform, and the area representing the calculated difference index (DI) is highlighted in gray. a.u. - arbitrary units.

Each pair of reliable (not rejected) ICP and C_a_BV pulses were then normalized to values range of 0–1 and synchronized with regard to the beginning of the ascending slopes (the minimum values at the beginning of the pulses). Finally, the difference index (DI) was calculated as the sum of absolute differences between values of ICP and C_a_BV at each time instant starting from the pulse onset point to the end of the C_a_BV pulse (see Fig. 4). Custom-written Python scripts were used for all signal processing tasks.

### 2.4 Statistical analysis

Non-parametric tests were used for statistical analysis as the majority of variables did not meet the normality assumption tested by the Shapiro–Wilk test. Wilcoxon signed–rank test was used to examine the differences in physiological signals and DI between ICP plateau phase and the corresponding baselines. Significance level of 0.05 was chosen for all analyses. Calculated values of physiological signals and indices are presented using the median and first and third quartiles unless stated otherwise.

## 3 Results

15 ICP plateau waves recorded in 11 males and 4 females (median age: 23 years [Q1–Q3: 17–28 years]) were studied. The values of physiological signals averaged over the baseline and plateau periods are presented in Table 1.

**Table 1:** Medians, first (Q1) and third (Q3) quartiles of physiological signals during baseline and plateau phase of the plateau wave with Wilcoxon signed–rank test *p* values. ICP – intracranial pressure, CBFV – cerebral blood flow velocity, ABP – arterial blood pressure. n.s. – result not statistically significant.

Signal	Baseline		Plateau phase		*p* value
	Median	Q1 – Q3	Median	Q1 – Q3	
ICP [mm Hg]	19.4	18.2–23. 4	42.7	36.5–45.1	<0.001
CBFV [cm/s]	44.2	34.8–69.5	32.9	24.7–68.2	0.002
ABP [mm Hg]	93.9	89.2–102.8	93.9	90.6–101.0	n.s.

**Fig. 5:**
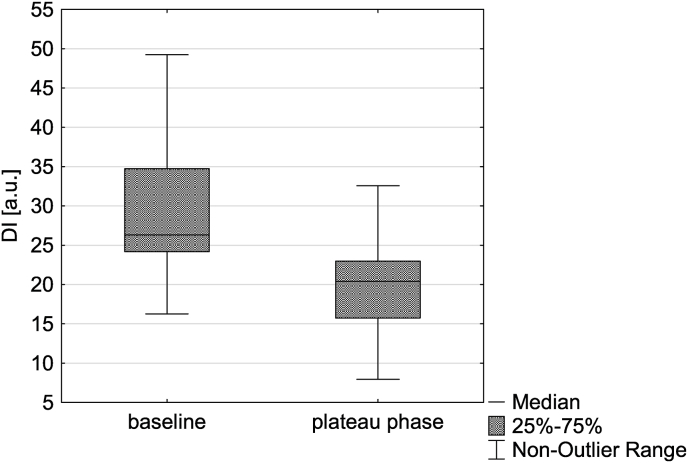
Comparison of the difference index (DI) between the baseline period and the plateau phase of intracranial pressure plateau wave.

**Fig. 6:**
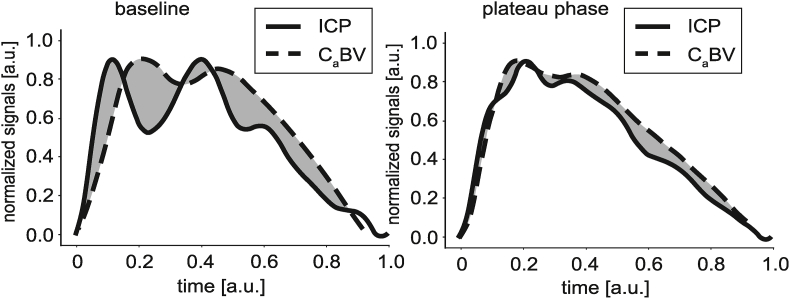
Illustrative examples of intracranial pressure (ICP) and cerebral arterial blood volume (C_a_BV) pulses recorded in a single patient during baseline and plateau period of the plateau wave. The gray area indicates the difference index (DI) between the pulses which is visibly smaller during the plateau phase as the ICP pulse waveform becomes more rounded compared to the baseline triphasic shape. a.u. - arbitrary units.

A total of 8967 pairs of ICP and C_a_BV pulses were analyzed (5149 at baseline and 3818 at plateau phase). The DI was lower during plateau phase of the ICP plateau wave than at baseline (p < 0.001, see Fig. 5), indicating that the similarity between the waveforms increased. An illustrative example of the change in similarity between individual ICP and C_a_BV pulses during the periods of baseline and plateau phase in a single patient is presented in Fig. 6.

## 4 Discussion

In this study we performed a quantitative analysis to investigate the similarity between the shape of cardiac-related pulses of ICP and C_a_BV in TBI patients during episodes of ICP plateau waves. Our findings revealed that the area between the corresponding ICP and C_a_BV pulse waveforms decreases during the occurrence of ICP plateau waves. This observation provides support for the hypothesis that during 10.13039/100011042ICP plateau waves the shape of 10.13039/100011042ICP pulses becomes more similar to that of C_a_BV pulses. A comparable observation was previously made by Carrera et al. (2010) who noted that changes in the pulsatility of C_a_BV predominantly influenced the amplitude of ICP pulses in TBI patients. Our study further advances this research by focusing not on the amplitude of ICP pulses but on their shape and providing a quantitative measure for assessing their similarity to C_a_BV.

Pulsatile changes in cerebral blood flow lead to pulse alterations in C_a_BV that in turn result in the expansion of cerebral arterial walls. Vessel expansion is then transmitted to the surrounding brain tissue and cerebrospinal fluid, thereby influencing the shape of the ICP pulse waveform. This transmission is likely intensified during ICP plateau waves due to an increase in cerebral blood volume caused by vasodilation in response to a drop in ABP. Moreover, TBI patients often experience disturbances in cerebral autoregulation which can further amplify this phenomenon. Previous studies have also observed alterations in the shape of ICP pulse waveforms toward more pathologically rounded pulses in TBI patients, even at mean ICP levels below 20 mm Hg (Hawthorne and Piper, 2014; Kazimierska et al., 2021; Mataczynski et al., 2022; Uryga et al., 2022), which suggests that cerebrospinal compliance is often reduced in this population. When intracranial compensatory reserve is depleted (i.e., cerebrospinal compliance is low), pulsatile changes in C_a_BV appear to be more directly transmitted to the ICP signal. During the plateau wave, intracranial compliance decreases, thus making this transmission even more effective.

It was previously demonstrated that the compliance of the cerebral arterial bed increases during plateau waves (Kim et al., 2009). The results of this study indicate that the C_a_BV pulse is a dominant factor that shapes the ICP pulse during plateau waves. These observations support those made by Risberg, Lundberg, and Ingvar (Risberg et al., 1969), who proposed that the increase in 10.13039/100011042ICP during plateau waves is driven by the increase in C_a_BV. Moreover, plateau waves have been observed in various pathophysiological conditions, including craniosynostosis (Renier et al., 1982), benign intracranial hypertension (Hayashi et al., 1991), head injury (Rosner and Becker, 1984), subarachnoid hemorrhage (Hayashi et al., 1984), brain tumors and acute hydrocephalus (Hayashi et al., 1987). These conditions are all related to disturbances in intracranial volume, which leads to a decrease in cerebrospinal compliance. Consequently, we hypothesize the elevated ICP observed during the plateau phase may be associated with an increase in C_a_BV under conditions of low cerebrospinal compliance. This implies that in individuals with high cerebrospinal compliance, a comparable change in C_a_BV would result in a lower increase in ICP, which may not be classified as a plateau wave.

In recent years, there has been growing interest in non-invasive estimation of ICP, and numerous studies have been conducted in this area. The latest solutions allow for monitoring of surrogates of the ICP signal with pulse waveform analysis (Brasil et al., 2021), which are likely driven by changes in C_a_BV. This could potentially be useful in detecting increases in ICP. Further research is required to confirm these hypotheses.

Certain limitations of this study should be noted. It was performed in a small number of patients due to the low probability of capturing the occurrence of ICP plateau wave during short-term TCD monitoring. Therefore, these results should be treated as preliminary and further research is needed to confirm our observations. Another limitation is that all signals underwent up-sampling from 50 Hz to 200 Hz to enhance their temporal resolution followed by low-pass filtering with a cut-off frequency of 12 Hz. This filtering process might have had a slight effect on the shape of the pulses and resulting similarity measures. Nevertheless, if any such influence did occur, it was consistent across all pulses and can be replicated reliably. The changes in heart rate may also have impacted the results of this study due to either shortening or lengthening of the pulses. We plan to normalize the pulse waveforms along the time axis to exclude this influence in further studies. Finally, the estimation of C_a_BV based on TCD measurement relies on the assumptions that: (1) the cerebral blood outflow can be approximated by a mean value of cerebral blood inflow; and (2) cerebral blood flow can be replaced with CBFV, i.e. that the diameter of the insonated artery is constant over a single cardiac period. The equation for C_a_BV is normalized by the cross sectional area of the insonated artery and it has cm units instead of cm^3^. Therefore, C_a_BV estimated this way can be used to observe relative changes in C_a_BV in a single patient over time, but the mean value cannot be compared between patients. Moreover, the constant flow forward model of brain blood circulation has not been validated in large populations and requires further studies to confirm its reliability. However, it has been successfully used in comparable studies, including investigations related to ICP plateau waves in TBI patients (Carrera et al., 2010; Kim et al., 2009).

## 5 Conclusions

Our study revealed that during ICP plateau waves, there is an increase in the similarity between the waveform shapes of ICP and C_a_BV pulses accompanied by a significant reduction in the area between the corresponding pulse contours. These findings strongly indicate that changes in C_a_BV pulsatility primarily dictate the shape of ICP pulses during ICP plateau waves. The presented methodology may have clinical applications in quantifying the similarity between ICP and C_a_BV waveforms, potentially offering a non-invasive approach to the detection of plateau waves.

## Funding

This study was supported by the 10.13039/501100004442National Science Centre, Poland (grant no. UMO-2019/35/B/ST7/00500). MC was supported by the 10.13039/501100008530ERDF (European Regional Development Fund) via the 10.13039/100013276Interreg France (Channel) England Programme. MC is supported by 10.13039/501100000272NIHR, Cambridge Centre and Med-Tec MIC cooperative.

## Disclosures

MC has a financial interest in part of the licensing fee of ICM + software used for signal recording and analysis. The other authors have nothing to disclose.

## Author contributions

**AZ**: methodology (lead); formal analysis (equal); software (supporting); visualization (lead); writing – original draft (lead). **MK**: conceptualization (lead); methodology (supporting); supervision; formal analysis (supporting); writing – original draft (supporting). **AK**: methodology (supporting); software (lead); writing – review & editing (equal). **MC**: data collection, writing – review & editing (equal).

## Declaration of competing interest

The authors declare the following financial interests/personal relationships which may be considered as potential competing interests: Arkadiusz Ziolkowski reports financial support was provided by National Science Centre Poland. Magdalena Kasprowicz reports financial support was provided by National Science Centre Poland. Agnieszka Kazimierska reports financial support was provided by National Science Centre Poland. Marek Czosnyka reports financial support was provided by European Regional Development Fund. Marek Czosnyka reports support was provided by National Institute for Health and Care Research , Cambridge. Marek Czosnyka has patent licensed to Part of the licensing fee of ICM + software.
